# Implementing Artificial Intelligence in Family Medicine: Challenges and Limitations

**DOI:** 10.7759/cureus.75518

**Published:** 2024-12-11

**Authors:** Paraskevi F Katsakiori, George C Kagadis, Francesk Mulita, Markos Marangos

**Affiliations:** 1 Department of Family Medicine, Health Center of Akrata, Akrata, GRC; 2 Faculty of Medicine, University of Patras, Rion, GRC; 3 Department of Surgery, General University Hospital of Patras, Patras, GRC; 4 Department of Surgery, General Hospital of Eastern Achaia - Unit of Aigio, Aigio, GRC

**Keywords:** artificial intelligence, family medicine, general practice, medical education, primary health care

## Abstract

General practice/family medicine is a primary healthcare discipline that focuses on providing comprehensive, patient-centered care to individuals and families across their lifespan. This study aimed to assess the implementation of artificial intelligence (AI) in general practice/family medicine regarding the 12 characteristics of its discipline as described in the recently revised European Definition of General Practice/Family Medicine. AI has the potential to revolutionize family medicine practice by improving the accuracy, efficiency, and effectiveness of various clinical tasks. From diagnostic decision-making to chronic condition management and clinical workflow optimization, AI-powered tools offer a range of benefits for patients and clinicians. However, appropriate education and training of the primary healthcare providers are deemed necessary to maximize benefits and minimize potential pitfalls. To ensure efficient and safe integration of AI in medical practice, important implications regarding privacy, malpractice, bias, autonomy, and overtreatment should also be addressed. AI application should be treated as a practical, assistive tool for the clinician, that can hardly take over decision-making and cannot replace the doctor-patient relationship.

## Introduction and background

Artificial intelligence (AI) is a group of computing techniques used to develop computer systems that can process information, carry out tasks (decision-making, problem-solving, learning), and react in a human-like manner [1,2]. Since Alan Turing came up with the idea of *thinking machines*, all efforts have been made toward developing machine learning tools that can resemble human cognitive functions and be used in several fields including healthcare. In this direction, AI has emerged as a promising tool for enhancing the delivery of healthcare services, including primary healthcare. More specifically, AI can be applied in several terms of healthcare such as administrative applications, virtual patient care, medical and imaging diagnostics, medical research and drug delivery, patient engagement and compliance, and rehabilitation [3].

General practice/family medicine is a primary healthcare discipline focusing on providing comprehensive, person-centered care to individuals and families across their lifespans [4]. The field is characterized by its holistic approach to healthcare, which involves understanding and addressing the social, psychological, and environmental factors that can influence health outcomes. Besides, in most countries, general practitioners/family doctors are the first to see the patient. They are expected to diagnose patients early and precisely while minimizing the cost of healthcare by avoiding excessive testing and/or specialist referral.

In general, AI and medical care display a two-way relationship as each partner needs the other one to evolve [5,6]. For this reason, a match between AI effect and clinical needs should be guaranteed as AI has the potential to change medical care by improving the accuracy, efficiency, and effectiveness of various clinical tasks such as diagnosis, treatment, and monitoring of chronic conditions. Routine task completion, decision support, patient education and empowerment, as well as the analysis of practice data and existing evidence, are some of the fields that AI applications could help [7]. Nevertheless, some ethical, medical, legal, and social implications of AI use in primary healthcare are raised [7].

In this study, the implementation of AI in general practice/family medicine was assessed regarding the 12 characteristics of its discipline as described in the recently published European Definition of General Practice/Family Medicine (WONCA Europe) in 2023 [4].

## Review

The first approach

In most countries, general practitioners/family doctors are normally the first medical doctors to see an individual who deals with a health problem and approach the healthcare system “regardless of the age, sex, or any other characteristic of the person concerned” [4]. AI has the potential to revolutionize family medicine practice by improving the accuracy, efficiency, and effectiveness of various clinical tasks. Additionally, the integration of machine learning tools in clinical practice can theoretically decrease the pressure on the medical system by triaging patients and informing them if they must seek help in an emergency manner [8]. For example, originally chatterbot, a software application or web interface aiming to imitate human conversation through text or voice interactions, can be used to triage patient requests and schedule appointments, reducing the workload on front-line staff, and freeing up time for clinicians to focus on more complex cases. In contrast, patients’ records are sensitive and confidential; thus, data accessibility is a composite problem [3,9]. Potential bias in AI systems has also been described regarding health equity [10-12]. In contrast, Alami et al. proposed that AI can potentially help in the reduction of health inequalities, especially in low- and middle-income countries, and can ensure access to high-quality and affordable health care for all [13]. Regardless of the successful performance of machine learning tools in medical practice, the aforementioned concerns, especially in under-trained practitioners, overshadow the acceptance and integration of AI in primary health care [14]. In 2018, Blease et al. conducted a qualitative survey of general practitioners in the United Kingdom and concluded that clinicians showed optimism but skepticism as well on the idea of replacing humans with AI in health care in certain tasks [15].

Coordinating care and working with other professionals 

General practitioners/family doctors should ensure “efficient use of health care resources through co-ordinating care, working with other professionals in the primary care setting, and by managing the interface with other specialties taking an advocacy role for the patient when needed...” [4]. In this direction, primary health care providers rely basically on patient’s history and clinical examination as they have limited immediate access to laboratory tests or imaging diagnostics. However, referrals should be appropriate, and testing should be decreased to the necessary so that the cost is minimized. The development and use of accurate machine learning classifiers and clinical prediction models could be of great assistance in primary healthcare. Ellertsson et al. developed such a machine learning classifier and evaluated its accuracy on primary headache diagnoses in primary health care [16]. The classifier outperformed the general practitioners on all metrics studied (sensitivity, positive predictive value, Matthews correlation coefficient, Sharpley additive explanations values, receiver operating characteristic [ROC] curve, and area under the ROC curve) except for specificity. Besides, a key challenge remains to develop machine learning tools that align with people’s interests. Another challenge is the need for effective communication and collaboration between clinicians and machine learning tools [17]. Technicians should use and establish a larger, more comprehensive database to maximize accuracy, thus increasing the trust of health workers and patients in AI [8]. However, human functions such as teamwork and team management are difficult to achieve since machines cannot form bonds with humans [2,3].

Person-centered approach

General practice/family medicine approaches first the individual and second the illness in everyone. In other words, it “develops a person-centered approach”; considers the beliefs, fears, expectations, and needs of the individual; and is “orientated to the individual, his/her family, and their community” [4]. In contrast, AI technology focuses on the diagnosis of a disease usually irrespective of the social and psychological characteristics of the patient [18,19]. Although it seems easy to teach and train a machine on the predictive values of various symptoms, it is even impossible to develop a system that can treat humans with empathy, care, and compassion, while considering their personal characteristics (cultural, psychological, etc.) [9,18]. Without a patient-centered orientation as well as practitioners’ focus on value and personal relationships, the use of AI can cause more harm rather than benefit to the community [5]. Therefore, AI applications should assess and adapt to patients’ and doctors’ preferences. 

Patient empowerment

Family medicine plays a strategic role in promoting *patient empowerment* [4]. Nonetheless, in the last decade, medical knowledge and clinical expertise seem to slip the doctor’s office. Patients can review on the internet or most recently developed machine learning tools like ChatGPT [2,5,20]. However, improving health literacy and providing patients with knowledge and skills to interpret the information found in the network are deemed necessary [12,20]. Patient empowerment and self-management contribute to achieving the goals set during treatment and ameliorating patient’s quality of life, especially when dealing with chronic diseases. However, to ensure these, continuous patient education is needed based on both strong therapeutic relationships and a person-centered approach [12]. 

Overtime relationship and effective communication

All medical doctors should communicate effectively with their patients. Especially in the case of general practice/family medicine, a long-standing relationship is being established over time and mostly throughout the whole of their life. Each visit and each contact are a part of “an evolving story” [4]. Clinician’s communication skills are of great value in the establishment of the therapeutic, doctor-patient relationship [11]. However, when it comes to the use of AI in medical practice and especially in primary health care, we should not forget that computers are practitioners’ partners and not opponents [5,21]. In other words, in family medicine, personal relationships have always been, are, and will always be of great importance in the establishment of the therapeutic relationship [5,9]. Patients trust their general practitioner, and they accept to share health data with their doctor. In the study of Mikkelsen et al., patients showed a positive attitude toward AI applications with the guarantee that their implementation would be done in a way that preserves the therapeutic relationship and would be used to help primary health doctors [22]. Although machine learning tools can be used to replace less complex but time and labor-consuming tasks in everyday clinical practice, there is always the possibility they may compromise the development of a successful doctor-patient relationship if used hugely [8,21,23]. It is the touch, the expressions, the smile, and the emotions that form part of effective communication and these cannot be provided by AI.

Longitudinal continuity of care

Primary health care is and must be constant and continuing “from birth (and sometimes before) until death (and sometimes afterward)” [4]. In most countries, primary health care is the largest healthcare delivery platform whilst on the other hand, the volume of information that family doctors manage will continuously grow probably in the cost of the available time for patient care. The wide use of electronic medical record systems over the last decades has helped medical doctors handle the great volume of information and can surely provide AI applications with big data. However, assessing the impact of AI applications on continuity and coordination of care may be difficult [23]. All the information gained over the years is gathered and presented in the medical record. AI-powered tools can help clinicians track patient data over time and provide personalized treatment based on the patient's unique health profile. This can help patients achieve better outcomes and reduce the risk of complications associated with their conditions. More specifically, Digital Twins (DTs) are dynamic, virtual representations of physical systems, and/or processes that are dynamically updated with real-time data from their physical equivalents. These models integrate sensor data, and machine learning to simulate, monitor, and foretell the behavior of the physical entity they represent [24].

Decision-making process

Diagnosis is a decision-making process that is influenced not only by the symptoms, signs, and laboratory tests that a patient exhibits but it also has to do with the characteristics of the patient, the community, and the place he lives in. The prevalence and incidence of an illness varies according to the community and the place where medical care is provided. For instance, serious disease presents less frequently in primary health care than in the hospital [4]. To successfully diagnose an illness, general practitioners/family doctors should use a specific probability decision-making process enriched by the knowledge of patients and the community [16]. Unfortunately, training datasets for AI applications are not always representative of specific populations although neural networks can continuously learn and adapt to a particular patient population or individual treatment preferences [23,25-28]. In other words, machine learning algorithms can be trained to analyze patient data and identify patterns that can help clinicians make more accurate diagnoses. This can be useful when a patient presents with a complex set of symptoms that can be difficult to diagnose based on clinical intuition alone. However, the ethical matter of who is responsible for possible diagnostic errors that may occur during the use of an AI tool has to be addressed [8]. Clinicians need to understand how AI algorithms work and what their limitations are to ensure they can interpret and act on the recommendations provided by these tools appropriately. Similarly, machine learning tools need to be designed to be transparent and interpretable so that clinicians can trust their output and use it to confirm their clinical decision-making [6,7,17,19]. 

Acute and chronic health problems

Many times, patients approach the healthcare system with more than one complaint and/or more than one illness [4]. The number of complaints or illnesses increases with age and general practitioners/family doctors should set priorities while considering patient’s wishes and needs. In other words, family medicine “manages simultaneously both acute and chronic health problems of individual patients” [4]. AI can be useful in family medicine practice in the management of chronic conditions, such as diabetes, hypertension, and heart disease. AI decision support systems may assist clinicians in making more informed treatment decisions by providing access to the most current evidence-based guidelines and best practices. However, it's interesting to question who bears the responsibility for potential diagnostic errors when an AI tool is involved in diagnosing a patient [8,29]. While a physician misdiagnosis may affect a single patient, an error by an AI tool could have far-reaching consequences, impacting thousands of patients. Therefore, serious concerns regarding privacy, malpractice, and overtreatment should be addressed before the safe and effective integration of machine learning tools into clinical practice. In addition, the assurance of an unbiased AI system is crucial. Identifying and mitigating bias in unsupervised algorithms must be a priority in all AI uses [23]. Moreover, the business model behind AI systems and the *black box* nature of the incorporated algorithms may contribute to a lack of trust among healthcare practitioners.

Diagnosis at an early stage

Family physicians/general practitioners manage “illness which presents in an undifferentiated way at an early stage in its development, which may require urgent intervention” [4]. As the patient usually presents to the family practice at the onset of symptoms, important decisions should be taken based on limited information [4]. Early signs of a disease are usually nonspecific and can be found in several diseases. In this case, a top priority is to exclude any serious outcome and let the time pass and review later when clinical signs develop further. Toward this direction, efficient and safe risk management is of great value. Some of the most significant benefits of AI integration in medical practice are the efficacy, accuracy, and precision of the diagnosis [18]. In addition, machine learning tools ensure better monitoring and save money [18]. However, one of the main challenges to be addressed is the need for robust data infrastructure and interoperability standards to ensure that AI algorithms can access and analyze patient data from a variety of sources. This requires significant investment in healthcare information technology (IT) systems and data governance processes. Despite methodological and computational advances in AI over the last few years, few of them can be translated into routine clinical practice as they may lead to unnecessary treatment against evidence-based guidelines [23].

Promoting the health and well-being of patients and ecosystems

Early interventions should be appropriate and effective; otherwise, they may cause harm or increase costs [4]. Predictive modeling is, by far, the most common use of AI in general practice/family medicine [2]. For example, AI has been used for the detection of high-risk patients with cardiovascular disease. Additionally, the main aspects of primary care practice, including disease prevention and health promotion, can be enhanced by the implementation of machine learning tools. In their scoping review, Willis et al. demonstrated meaningful improvement in both clinical and nonclinical outcomes using preventive digital health interventions [30]. 

Responsible for the health of the community and environment

General practitioners/family doctors take care of both the individual patient and the community overall. Expert systems, natural language processing, and machine learning seem to be the most used in community-based primary health care [31]. However, the implementation of machine learning tools in community-based primary healthcare is complicated by the variability of patients’ data, age, cognitive abilities, etc. [31]. Additionally, in the recently published edition of the European Definition of General Practice/Family Medicine, the *One Health* concept of the World Health Organization was integrated [4]. Optimizing people’s health should balance with the health of the animals and the environment. 

Physical, psychological, social, cultural, and existential dimensions

In clinical practice, otherwise, successful therapeutic interventions can prove unsuccessful in a certain patient as they were probably designed without considering specific characteristics of the patient (social, psychological, cultural, etc.). Reading the emotions and body language of each patient provides practitioners with significant information regarding the patient’s needs and attitude and plays an important role in the individualization of the therapeutic plan and the success of patient care. In the scoping review by Rahimi et al., the authors aimed to evaluate the published literature that has tested or implemented AI to facilitate shared decision-making [32]. Six peer-reviewed publications were included in the study, three of them focused on primary care. The authors observed a lack of attention to patients’ values and preferences. Although participatory co-design and iterative implementation have been proposed to overcome such barriers, the lack of human touch, empathy, and emotional intelligence may hamper the benefits of AI applications in medical care [7,19].

## Conclusions

All 12 general practice/family medicine characteristics, ranging from diagnostic decision-making to chronic condition management and clinical workflow optimization, are expected to be positively affected by AI-powered tools as they offer a range of benefits for both patients and clinicians. However, appropriate medical education and training of primary healthcare providers are deemed necessary to maximize benefits and minimize potential harm. The participation of the final user (i.e., medical doctor, nurse practitioner, etc.) in machine learning tools’ design will surely help in the uneventful integration of novel technology in medical practice. Additionally, specific guidelines along with specific legislation protecting healthcare practitioners when using AI tools are needed to secure certain issues such as the liability for *black box* approaches of AI or the liability of the clinician. In conclusion, to ensure efficient and safe integration of AI in medical practice, important implications regarding privacy, malpractice, bias, autonomy, and overtreatment should be assessed in more detailed research where the opportunities of AI tools will be studied and tailored to the needs of primary healthcare practice.

## References

[1] Bajgain B, Lorenzetti D, Lee J, Sauro K (2023). Determinants of implementing artificial intelligence-based clinical decision support tools in healthcare: a scoping review protocol. BMJ Open.

[2] Irfan F (2021). Artificial intelligence: help or hindrance for family physicians?. Pak J Med Sci.

[3] Al Kuwaiti A, Nazer K, Al-Reedy A (2023). A review of the role of artificial intelligence in healthcare. J Pers Med.

[4] WONCA WONCA (2023). The European Definition of General Practice/Family Medicine. European Academy of Teachers in General Practice (Network within WONCA Europe.

[5] Liaw W, Kakadiaris IA (2020). Artificial intelligence and family medicine: better together. Fam Med.

[6] Kueper JK, Terry AL, Zwarenstein M, Lizotte DJ (2020). Artificial intelligence and primary care research: a scoping review. Ann Fam Med.

[7] Terry AL, Kueper JK, Beleno R (2022). Is primary health care ready for artificial intelligence? What do primary health care stakeholders say?. BMC Med Inform Decis Mak.

[8] Li L (2019). Artificial intelligence and diagnosis in general practice. Br J Gen Pract.

[9] Padhan S, Mohapatra A, Ramasamy SK, Agrawal S (2023). Artificial intelligence (AI) and robotics in elderly healthcare: enabling independence and quality of life. Cureus.

[10] Abràmoff MD, Tarver ME, Loyo-Berrios N (2023). Considerations for addressing bias in artificial intelligence for health equity. NPJ Digit Med.

[11] Wang JX, Somani S, Chen JH, Murray S, Sarkar U (2021). Health equity in artificial intelligence and primary care research: protocol for a scoping review. JMIR Res Protoc.

[12] Darcel K, Upshaw T, Craig-Neil A (2023). Implementing artificial intelligence in Canadian primary care: barriers and strategies identified through a national deliberative dialogue. PLoS One.

[13] Alami H, Rivard L, Lehoux P (2020). Artificial intelligence in health care: laying the foundation for responsible, sustainable, and inclusive innovation in low- and middle-income countries. Global Health.

[14] Liaw W, Kueper JK, Lin S, Bazemore A, Kakadiaris I (2022). Competencies for the use of artificial intelligence in primary care. Ann Fam Med.

[15] Blease C, Bernstein MH, Gaab J (2018). Computerization and the future of primary care: a survey of general practitioners in the UK. PLoS One.

[16] Ellertsson S, Loftsson H, Sigurdsson EL (2021). Artificial intelligence in the GPs office: a retrospective study on diagnostic accuracy. Scand J Prim Health Care.

[17] Hah H, Goldin DS (2021). How clinicians perceive artificial intelligence-assisted technologies in diagnostic decision making: mixed methods approach. J Med Internet Res.

[18] Amisha Amisha, Malik P, Pathania M, Rathaur VK (2019). Overview of artificial intelligence in medicine. J Family Med Prim Care.

[19] Summerton N, Cansdale M (2019). Artificial intelligence and diagnosis in general practice. Br J Gen Pract.

[20] Schulz PJ, Nakamoto K (2013). Patient behavior and the benefits of artificial intelligence: the perils of "dangerous" literacy and illusory patient empowerment. Patient Educ Couns.

[21] Upshaw TL, Craig-Neil A, Macklin J, Gray CS, Chan TC, Gibson J, Pinto AD (2023). Priorities for artificial intelligence applications in primary care: a Canadian deliberative dialogue with patients, providers, and health system leaders. J Am Board Fam Med.

[22] Mikkelsen JG, Sørensen NL, Merrild CH, Jensen MB, Thomsen JL (2023). Patient perspectives on data sharing regarding implementing and using artificial intelligence in general practice - a qualitative study. BMC Health Serv Res.

[23] Liyanage H, Liaw ST, Jonnagaddala J, Schreiber R, Kuziemsky C, Terry AL, de Lusignan S (2019). Artificial intelligence in primary health care: perceptions, issues, and challenges. Yearb Med Inform.

[24] Papachristou K, Katsakiori PF, Papadimitroulas P, Strigari L, Kagadis GC (2024). Digital twins’ advancements and applications in healthcare, towards precision medicine. J Pers Med.

[25] Papadimitroulas P, Brocki L, Christopher Chung N (2021). Artificial intelligence: deep learning in oncological radiomics and challenges of interpretability and data harmonization. Phys Med.

[26] Plachouris D, Tzolas I, Gatos I (2021). A deep-learning-based prediction model for the biodistribution of (90) Y microspheres in liver radioembolization. Med Phys.

[27] Eleftheriadis V, Savvidis G, Paneta V, Chatzipapas K, Kagadis GC, Papadimitroulas P (2023). A framework for prediction of personalized pediatric nuclear medical dosimetry based on machine learning and Monte Carlo techniques. Phys Med Biol.

[28] Plachouris D, Eleftheriadis V, Nanos T (2023). A radiomic- and dosiomic-based machine learning regression model for pretreatment planning in (177) Lu-DOTATATE therapy. Med Phys.

[29] Bhattacharya S, Pradhan KB, Bashar MA (2019). Artificial intelligence enabled healthcare: a hype, hope or harm. J Family Med Prim Care.

[30] Willis VC, Thomas Craig KJ, Jabbarpour Y (2022). Digital health interventions to enhance prevention in primary care: scoping review. JMIR Med Inform.

[31] Abbasgholizadeh Rahimi S, Légaré F, Sharma G (2021). Application of artificial intelligence in community-based primary health care: systematic scoping review and critical appraisal. J Med Internet Res.

[32] Abbasgholizadeh Rahimi S, Cwintal M, Huang Y (2022). Application of artificial intelligence in shared decision making: scoping review. JMIR Med Inform.

